# Evaluation of canonical Hedgehog signaling pathway inhibition in canine osteosarcoma

**DOI:** 10.1371/journal.pone.0231762

**Published:** 2020-04-29

**Authors:** Vincent E. Baldanza, Anita Rogic, Weiwei Yan, Corri B. Levine, Roy A. Levine, Andrew D. Miller, Angela L. McCleary-Wheeler

**Affiliations:** 1 Department of Clinical Sciences, College of Veterinary Medicine, Cornell University, Ithaca, New York, United of States America; 2 Department of Veterinary Medicine and Surgery, College of Veterinary Medicine, University of Missouri, Columbia, Missouri, United of States America; 3 Department of Molecular Medicine, College of Veterinary Medicine, Cornell University, Ithaca, New York, United of States America; 4 Department of Biomedical Sciences, Section of Anatomic Pathology, College of Veterinary Medicine, Cornell University, Ithaca, New York, United of States America; Centre for Applied Medical Research, University of Navarra, SPAIN

## Abstract

Canine osteosarcoma (OSA), the most common canine primary bone malignancy, has a highly aggressive biologic behavior. Despite current standard of care therapies, including amputation and adjuvant chemotherapy, most dogs still succumb to metastatic disease. Further investigations into molecular mechanisms and pathways driving OSA are needed to improve therapeutic options. The Hedgehog (HH) cell-signaling pathway has demonstrated involvement in human OSA. Several studies in canine OSA have found changes in expression of some HH pathway genes and demonstrated a role for HH transcription factors. However, the role of this pathway as well as the translational value of its targeting in canine OSA are still undefined. The objectives of this study were to determine the expression of HH components directly in canine OSA tissues and to evaluate the biologic impact of HH signaling inhibition in canine OSA cells. *In situ* hybridization was used to detect HH family mRNA expression in archived canine OSA tissues and revealed variable expression levels of these mRNAs in canine OSA tissues. The effect of a commercially available Smoothened inhibitor, vismodegib, was studied in established canine OSA cell lines. Alterations in cellular growth as well as assessment of downstream HH targets were evaluated. Although changes in cell growth were noted following Smoothened inhibition, inconsistent decreases in target gene expression were found. While treatment with vismodegib had a negative impact on canine OSA cell growth and viability, the mechanism remains unclear. Further studies are warranted to evaluate the clinical significance of canonical HH signaling in canine OSA.

## Introduction

Canine osteosarcoma (OSA) is an aggressive mesenchymal malignancy of bone that produces an extracellular osteoid matrix [1]. OSA is the most common skeletal malignancy of dogs [1, 2]. This tumor occurs primarily in older, large to giant breed dogs, and involvement of the appendicular skeleton represents about 75% of cases [1–5]. Canine OSA is biologically aggressive with destructive local behavior and high metastatic rates [1]. Local disease results in severe pain due to a combination of bone lysis and production. Hematogenous spread of neoplastic cells occurs early in the disease, and the lungs are the most common metastatic sites [2]. Though less than 15% of cases have radiographically detectable metastasis at diagnosis, 90% of patients die with metastatic disease within one year of diagnosis [6, 7]. Surgery alone is considered palliative with average survival times of 4–6 months as the metastatic component is not addressed [6]. Adjuvant chemotherapy with doxorubicin and/or platinum drugs is recommended to delay the onset of metastatic disease for patients undergoing surgery [6–14]. Though use of these agents significantly extends survival times to 10–12 months on average, the development of metastatic lesions eventually occurs in most patients [6–14]. Due to the stagnation in achievement of improved disease outcomes, novel therapeutic are needed.

Canine OSA parallels OSA in children in numerous aspects. It is proposed as a natural model for human OSA, which is the most common primary bone malignancy in children and represents 5% of all childhood cancers in the United States [15, 16]. The Hedgehog (HH) developmental signaling pathway has been studied in human OSA and contributes to the pathogenesis of human OSA [17–28]. Canonical HH signaling occurs through the 12-pass transmembrane receptor Patched (PTCH1), which normally maintains an inhibitory function over Smoothened (SMO), a 7-pass transmembrane receptor, in the absence of the HH ligands [18, 19]. Upon binding one of the HH ligands, including Sonic Hedgehog (SHH), Desert Hedgehog (DHH), or Indian Hedgehog (IHH), PTCH1 releases its inhibitory effect on SMO. This event leads to activation of the downstream cascade, with dissolution of an inhibitory complex containing Suppressor of Fused (SUFU), and concluding with the activation of the glioma-associated oncogene (GLI) zinc-finger transcription factors [18, 19].

In normal bone, the HH pathway tightly regulates growth and differentiation [20–22]. High expression levels of IHH and SHH are found in human OSA tumors and their microenvironment [23]. High expression levels of GLI2 correlated with a poor prognosis in human OSA patients and plays a role in proliferation, cell apoptosis, and sensitivity to chemotherapeutics [24–26]. GLI and SMO inhibition suppress proliferation of human OSA cells and prevent OSA growth *in vivo* [25, 27]. Hedgehog inhibition also prevented migration and metastasis of OSA in mouse models [28–32]. However, little research has been done regarding HH signaling in canine OSA. Gene expression profiling in canine OSA identified mRNA dysregulation of canine *Smo*, *Ptch1*, and *Dhh* in poor responders [15]. A recent study found inhibition of GLI function leads to decreased cell proliferation in canine OSA cell lines [31]. However, neither of these studies evaluated the expression patterns of HH genes at the cellular level in tissues nor the impact of upstream HH inhibition.

The goals of this study were to further characterize the expression patterns of HH pathway components in canine OSA and determine the impact of upstream HH inhibition, via Smoothened inhibition, on OSA biologic behaviors. We hypothesized that the HH pathway is active in canine OSA and that SMO inhibition negatively impacts canine OSA cell growth and viability *in vitro*.

## Materials and methods

### In situ hybridization

*In situ* hybridization (ISH) was performed on eleven archived, formalin fixed, paraffin embedded (FFPE) primary canine OSA samples identified from the Cornell University, New York State Diagnostic Center, Section of Anatomic Pathology archives. All samples were from appendicular OSA lesions amputated between the years 2012–2017. The ISH evaluated cellular expression of Smo, Ptch1, Gli1, Gli2, and IHH mRNA both in neoplastic and stromal cells comprising the tumor microenvironment. Specimens were sectioned at 5 μM (Accu-Edge Low Profile Microtome Blade, Sakura, Torrance, CA, 90501), mounted on positively charged Diamond White Glass microscope slides, and stored at -80°C. Alongside canine OSA tumor specimens, normal canine brain samples served as normal tissue positive control tissue, as these components of the Hedgehog signaling pathway are expressed in brain tissue.

ISH was performed using RNAscope Assay 2.5 HD Reagent Kit-Brown (Advanced Cell Diagnostics, 322300) and RNAscope 2.5 HD Detection Reagents-Brown (Advanced Cell Diagnostics, 322310) according to the manufacturer’s protocol. The target probes used were CI-Gli1 (XM_538247.5, region 2366–3475), CI-Gli2 (XM_014121472.1, region 2–1287), CI-Smo (XM_005628406.2, region 656–1519), CI-Ihh (XM_545653.5, region 847–1952) and CI-Ptch1 (XM_003638871.2, region 2423–3337). CI-PolR2A was used as a positive control, and DapB as a negative control. Briefly, FFPE sections were baked at 60°C for 1 hour, deparaffinized in xylene twice for 5 minutes, dehydrating in 100% ethanol twice for 1 minute, and dried at room temperature (RT) for 10 minutes. The slides were then pretreated with hydrogen peroxide for 10 minutes at RT, boiled in target retrieval reagents for 15 minutes and followed by protease treatment for 30 minutes at 40°C in a HybEZ Humidity Control Tray (Advanced Cell Diagnostics) with two rinses in distilled water between pretreatments. Target probes were then applied for 2 hours at 40°C in a HybEZ Humidity Control Tray followed by six amplification steps: AMP1 (30 minutes at 40°C), AMP2 (15 minutes at 40°C), AMP3 (30 minutes at 40°C), AMP4 (15 minutes at 40°C), AMP5 (30 minutes at RT) and AMP6 (15 minutes at RT). Slides were rinsed twice with wash buffer for 2 minutes between each AMP incubation. Finally, the mRNA signal was detected with DAB and the slices were counterstained with Gill’s hematoxylin and mounted in EcoMount mounting medium (Biocare Medical, EM897L). The level of expression was determined by visualization of the samples under 20X power. A score was assigned based upon positivity of cells evaluated as follows: + for <25% of cells having a positive signal; ++ for 25–50% of cells having a positive signal; +++ for 50–75% of cells having a positive signal; and ++++for >75% of cells having a positive signal.

### Cell lines and culture reagents

Three canine OSA cell lines were used in this study: Abrams (a kind gift from Dr. Timothy Fan–University of Illinois) [33, 34] and D-17 and HMPOS from the Cornell University Comparative Oncology Program (D-17 originally purchased from the American Type Culture Collection (ATCC CCL-183) [33, 34]. These lines have previously been used in numerous studies of canine OSA in the literature and are derived from metastatic OSA [33–35]. All three cell lines were maintained in Modified Eagle’s Medium (MEM, Corning, 10-010-CV) and supplemented with 10% Fetal Bovine Serum (Sigma, 4135), 1% penicillin-streptomycin (Corning, 30-002-CI), 1% MEM Nonessential Amino Acids (Corning, 20-025-CI), 1% MEM Vitamins (Corning, 20-020-CI), and 1% L-glutamine (Corning, 25-005-CI). All cell stocks were maintained on 150 mm tissue culture-treated plates (Corning Falcon, 353025) or 75 cm^2^ tissue culture-treated flasks (Corning, 430641U) at 37°C with 5% CO_2_. The medium and growth conditions were used for maintenance of cells as well as experiments. All cell lines were subjected to mycoplasma testing by Venor GeM Mycoplasma Detection Kit (Sigma, MP0035) and tested negative prior to starting experiments with re-testing throughout duration of the project. Cells were treated with various concentrations of the vehicle control (DMSO, Corning, 25-950-CQC) or the targeted Smoothened inhibitor vismodegib (Selleck Chemicals, S1082) [30]. The vismodegib was diluted into a stock concentration of 10 mM, divided into aliquots, and stored at -80°C until use.

### Cell proliferation assay

Cell proliferation was assessed via MTS assay using CellTiter 96 AQueous Non-radioactive Cell Proliferation Assay (Promega, G5421). MTS cell proliferation assays were performed on each cell line using multiple drug concentrations and time points. Abrams, D-17, and HMPOS cells were plated in 96-well tissue culture-treated plates (Corning, 3596) in triplicate at 1,000 cells per well and allowed to grow for 24 hours. After 24 hours, the medium was removed and replaced with 0 (DMSO vehicle control), 10, 20, 30, 40, and 50 μM of vismodegib. The cells were then allowed to incubate for 72hours. Respective media only (cell free) wells were used as a blank control. Following incubation for 72 hours with the treatments, assay reagents were added to the wells and allowed to incubate for 3 hours. Absorbance was read using multimodal plate reader (SpectraMax 190 Absorbance Microplate Reader, Molecular Devices or a Synergy H1, Biotek) at 490 nm wavelength. These experiments were performed in triplicate and the data obtained was utilized to calculate relative IC_50_ values for vismodegib for each cell line. The calculated relative IC_50_ values were then utilized for experimental treatment of cells in all other assays in this study.

### Clonogenic assays

Colony formation assays were performed using all three canine OSA cell lines. Cells were treated with vismodegib at the determined IC_50_ concentration, two-thirds of the determined IC_50_, one-third of the determined IC_50_, or vehicle control. Cells were plated on 60 mm tissue culture-treated plates (Corning Falcon, 353002) at 1,000 cells per dish for Abrams or 2,500 cells per dish for both D-17 and HMPOS. Cells were allowed to grow for 24 hours in normal growth medium. The medium was then removed and replaced with respective medium containing vismodegib or DMSO vehicle control. After 7 days of growth in respective treatments, plates were washed with PBS (Fisher, BP243820) and then allowed to fix in 100% methanol (Fisher, BP1105) for 12 minutes at room temperature, followed by a second methanol wash for 70 minutes. Colonies were then stained with 0.1% crystal violet (Sigma, V5265) in 95% ethanol (Fisher, BP2818) overnight before being washed with distilled water and allowed to dry. Colonies were defined as containing more than 50 cells through manual counting of each plate. Plates were counted manually, and both the plating efficiency and survival fractions were calculated. The plating efficiency was determined by dividing the total number of colonies formed in the DMSO treated plates by the number of cells seeded. The survival fractions were determined by dividing the number of colonies formed in the vismodegib treated plates by the number of cells seeded, and then normalized to the calculated plating efficiency. This allowed the comparison of the vismodegib treatment compared to that of vehicle control treatment. The clonogenic assays were performed in triplicate.

### RNA isolation and gene expression

Assessment of alterations in expression levels of canine HH pathway target genes (*Gli1*, *CyclinD1*, *Ptch1*, *Bmi1*, *Zeb1*, *and Snal1*) and our target for inhibition, *Smo*, were performed via reverse transcription quantitative polymerase chain reaction (RT-qPCR). RNA was isolated from OSA cells treated with vismodegib at their respective IC_50_ or with vehicle control. Cells were plated at 1 million (Abrams and D-17) or 2.5 million (HMPOS) cells per 100 mm tissue culture-treated plates (Corning, 430167) for 24 hours. The medium was then removed, and the cells were treated with vehicle control (DMSO) or their respective IC_50_ of vismodegib in growth medium for 72 hours of incubation. Total RNA was then isolated using the Trizol reagent (Invitrogen, 15596018) according to the manufacturer’s instructions. Briefly, medium was removed from the plates after incubation, 1 mL of Trizol was added, plates were scraped, and then total contents transferred to microcentrifuge tubes. Chloroform (Fisher, BP1145) was added to the microcentrifuge tubes, which were then shaken for 30 seconds and allowed to sit at room temperature for 10 minutes. Tubes were centrifuged at 4°C for 15 minutes at 12,000 rpm. The organic layer was transferred to a new microcentrifuge tube and washed with isopropanol (Fisher, BP2618500). Tubes were inverted 10 times, allowed to sit at room temperature for 10 minutes, and centrifuged at 4°C for 15 minutes at 12,000 rpm. The isopropanol was aspirated off, the RNA was washed with 75% ethanol, and the tube was centrifuged at 4°C for 5 minutes at 12,000 rpm. Ethanol was removed via pipetting, and the RNA pellet was allowed to dry for 15-minutes before adding nuclease-free water (Corning, 46-000-CV). Tubes were heated at 55°C for 10 minutes while shaking at 300 rpm to assist in RNA resuspension.

The RNA concentration of the samples was measured utilizing the microdrop function of an absorbance reader (SpectraMax 190, Molecular Devices or Synergy H1, Biotek). Samples were cleared of DNA using a DNase I kit (Invitrogen, 18068–015) according to the manufacturer’s instructions, in which 1 μg of RNA was mixed with 10X DNase I reaction buffer, DNase I, and nuclease-free water. This solution was incubated at room temperature for 15 minutes and the DNase was inactivated with 25 mM EDTA at 65°C for 10 minutes in a thermocycler (MasterCycler Nexus GSX1, Eppendorf). The DNase I treated RNA was then used to make cDNA using the SuperScript IV First-Strand Synthesis System (Invitrogen, 18091200) according to manufacturer’s instructions. The RNA obtained was mixed, centrifuged, and incubated with 50 μM oligo d(T)_20_ primer and 10 mM dNTP mix at 65°C for 5 minutes in a thermocycler. This product was then mixed, centrifuged, and incubated with RNA master mix, comprised of 5X SSIV buffer, 100 mM DTT, ribonuclease inhibitor, and SSIV Reverse Transcriptase at 55°C for 10 minutes, 80°C for 10 minutes, and then cooled to 4°C until removed from the thermocycler. Finally, the cDNA was incubated with RNase H at 37°C for 20 minutes.

Alterations in gene expression profiles were assessed with TaqMan assays (Life Technologies) following manufacturer’s instructions. Relative gene expression was shown in reference to a housekeeping gene, *Hprt1*. Briefly, the cDNA obtained above were added into optical 96-well plates (Applied Biosystems, 4306737) in triplicate for each cell line treated with vehicle control or vismodegib, as well as water-only samples, no reverse transcriptase samples, and no primer samples as negative controls. Master mix, comprised of nuclease-free water, the respective primer-probe (Taqman) assay, and Taqman Fast Advanced Master Mix (Applied Biosystems, 4444557), was added to the wells prior to covering the plate with an optical seal (Applied Biosystems, 4360954). Taqman (Applied Biosystems) gene expression assays for canine *Smo* (assay ID ARCE36R), *Gli1* (assay ID cf04230663_m1), *Cyclin D1* (assay ID cf02741728_m1), *Ptch1* (assay ID cf02690587_m1), *Bmi1* (assay ID cf02663120_m1), *Zeb1* (assay ID cf02725837_m1), and *Snai1* (assay ID cf02705362_s1) were utilized. The *Hprt1* gene (assay ID cf02626258_m1) was utilized as the housekeeping gene. An ABI QuantStudio3 thermocycler (Applied Biosystems) was used with the following TaqMan Assay Protocol Program: 95°C for 20 seconds, and 40 cycles of 95°C for 1 second and 60°C for 20seconds. Results were evaluated via the comparative delta-delta Ct method [36].

### Apoptosis assay

Assessment of apoptosis was accomplished via luminescent caspase-3/7 assay (ApoLive-Glo Multiplex Assay, Promega, G6410). For the caspase-3/7 assay, Abrams, D-17, and HMPOS cell lines were plated in triplicate in 96-well cell culture plates at concentration of 1,000 cells per well and allowed to grow for 24 hours. The medium was replaced with fresh medium containing their respective IC_50_ of vismodegib or DMSO vehicle control and allowed to incubate for 72 hours. Respective media only wells were used as a blank. All wells were treated with the viability reagent, mixed by orbital shaking at 300 rpm for 30 seconds, and incubated for 30 minutes at 37°C. Fluorescence was then measured using a fluorescence plate reader (SpectraMax M3, Molecular Devices) at a wavelength of 400_Ex_/505_Em_, to measure viable cells. Wells were then treated with Caspase-Glo 3/7 Reagent and mixed by orbital shaking at 300 rpm for 30 seconds. Plates were then incubated for 30 minutes at room temperature and luminescence was measured using the luminescent function of the plate reader (SpectraMax M3, Molecular Diagnostics) to measure caspase-3/7 reactivity, representing apoptotic cells. Luminescent values for apoptosis were divided by fluorescent values for viability to normalize via caspase-3/7/viability ratio, which was compared between vismodegib and vehicle control treated cells.

### Western blot

Western blot was performed to further visualize caspase and target proteins for assessment of apoptosis. Cells were plated at 1 million (Abrams and D-17) or 2.5 million (HMPOS) cells per 100 mm cell tissue culture-treated plates for 24 hours. Plates were then treated with vehicle control or their respective IC_50_ of vismodegib for 72 hours of incubation. Cells were then harvested for protein isolation by removing media and washing adherent cells with PBS. Plates were placed on ice and treated with RIPA lysis buffer (Tris Base, sodium chloride, 1% sodium deoxycholate, 1% Triton X-100, 0.1% SDS, and deionized water) containing protease inhibitors (Thermo Scientific, 78425). Cells were scraped into the buffer and transferred to microcentrifuge tubes. The media removed from the dishes containing non-adherent cells were centrifuged at 1,000 rpm for 5 minutes at 4°C and the media was aspirated off. The cell pellet was re-suspended in RIPA lysis buffer with protease inhibitor and combined with the harvested samples from adherent cells. Samples were transferred to TPX tubes (Diagenode, M-50050), incubated on ice for 15 minutes, subjected to 10 cycles of sonication at 30 sec each at 4°C using a Bioruptor UCD-300 (Diagenode), centrifuged at 4°C for 15 minutes at 12,000 rpm, and supernatants were transferred to a new microcentrifuge tube and kept on ice if run immediately or transferred to -80°C if not immediately used.

Harvested protein was quantified using the Pierce BCA Protein Assay (Thermo Scientific, 23225) according to kit protocol. Bovine serum albumin stock provided with the kit was used to make a standard curve, ranging from 0 to 2,000 μg/mL BCA. Standards were plated into a 96-well cell plate in triplicate, followed by protein samples at a 1:5 dilution with RIPA lysis buffer. Reagents were added to the wells and plates were incubated for 30 minutes at 37°C. Absorbance was then read using an absorbance reader (SpectraMax 190, Molecular Devices or BioTek Synergy H1) at 562 nm wavelength.

Twenty-five to fifty micrograms of protein from each sample was mixed 1:4 with 4X Laemmli Sample Buffer (BioRad, 1610747) and boiled for 5 minutes at 95°C. Sodium dodecyl sulphate polyacrylamide gel electrophoresis (SDS-PAGE) was performed using 16% tris-glycine SDS-PAGE gels and a XCell SureLock Mini-Cell electrophoresis system (ThermoFisher EI0002). Prepared samples were loaded in the wells, along with a standard protein marker ladder (BioRad, 1610374 or New England Biolabs, P7712 for caspase Westerns). Gels were electrophoresed at 100 V constant for one hour. The protein was transferred from the gel to polyvinylidene fluoride (PVDF) membranes (Millipore, IPVH00010) at 100V constant for one hour. Upon completion of transfer, the membranes were removed and blocked in 5% milk in tris-buffered saline/0.5% tween (TBS-T) solution for one hour at room temperature while shaking. The membrane was then washed three times for 10 minutes in TBS-T.

For the caspase Western blots, the membranes were incubated with anti-cleaved caspase-3 (Cell Signaling D175 #9661, 1:1,000) and anti-cleaved caspase-7 (Cell Signaling D6H1 #8438, 1:1,000) primary antibodies. Mouse Beta-actin (Sigma A2228, 1:10,000) was used as the housekeeping loading control. For evaluation of gene targets, the following antibodies were used: rabbit anti-GLI1 (Abcam ab49314, 1:400), rabbit anti-SMO (Abcam ab236465, 1:500), rabbit anti-Snai1 (Cell Signaling #3879. 1:500), and rabbit anti-Bmi1 (Cell Signaling #6964, 1:2000). Mouse alpha-tubulin (Sigma T9026, 1:5,000) was used as the housekeeping loading control. The antibody solutions were all prepared in TBST with 5% milk. Membranes were incubated overnight at 4°C while rocking, and then washed three times in 1X TBS-T. The membrane was then treated with appropriate secondary antibody, goat anti-rabbit or goat anti-mouse horseradish peroxidase conjugated (Millipore, AP132P and AP130, 1:5,000), in TBST with 5% milk for one hour at room temperature with rocking and then washed twice in TBS-T and once in TBS. SuperSignal West Dura chemiluminescent substrate (Thermo Scientific, 34076) was applied to the membranes for 5 minutes at room temperature. The membranes were then evaluated using radiographic film exposure (caspase blots) or a Kodak imaging system (all other blots).

### Statistical analysis

All experiments were performed in triplicate, with values reported as means with standard deviations, where applicable. Prism statistical software (GraphPad, v8.0) was used for data analysis. Determination of the IC_50_ was determined using nonlinear, four-point regression (with bottom constrained to 0) for the Abrams and HMPOS cell lines, while the D-17 cell line was evaluated with linear regression given the linearity of the results (R^2^ 0.9933). The IC_50_ was defined as the drug concentration that caused 50% metabolic activity when compared to the control treated cells. A one-way ANNOVA with Dunnett’s test for multiple comparisons was used for evaluation of the colony formation assays. The gene expression assays were evaluated with a multiple t-tests with Holm-Sidak correction for multiple comparisons. The cleaved caspase assay was evaluated with an unpaired t-test with Welch’s correction. Differences were considered significant if p < 0.05.

## Results

### Expression of HH pathway components vary in canine OSA tumor specimens

We sought to evaluate HH gene expression in canine OSA tumor tissue. To accomplish this, *in situ* hybridization (ISH) was performed on archived, formalin fixed, paraffin embedded primary canine appendicular OSA samples identified from the New York State Animal Health Diagnostic Laboratory and Department of Biomedical Sciences, Section of Anatomic Pathology archives between the years 2012–2017. The ISH evaluated cellular gene expression of *Ihh*, *Smo*, *Ptch1*, *Gli1*, and *Gli2* mRNA both in neoplastic cells and stromal cells comprising the tumor microenvironment.

**Fig 1:**
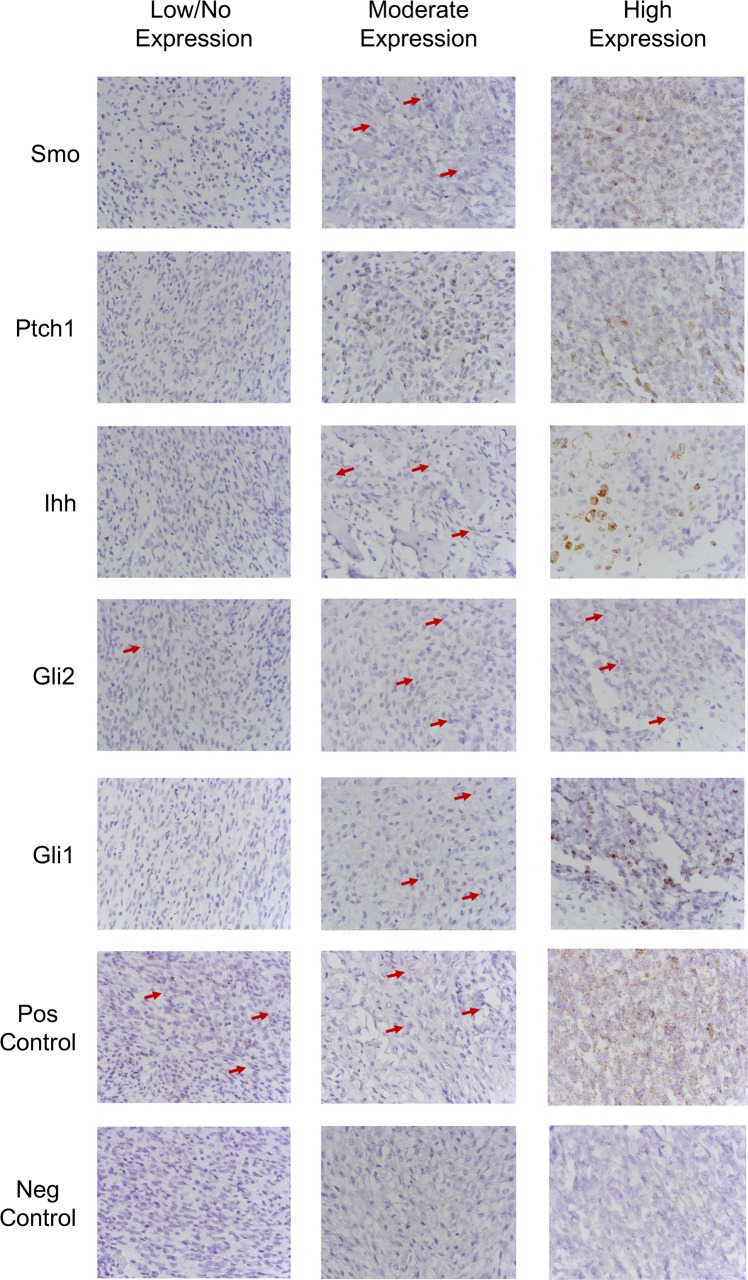
*In situ* hybridization for HH pathway components. Expression levels of HH pathway component mRNA, *Smo*, *Ptch1*, *Gli1*, *Gli2*, *and Ihh*, mRNA in canine OSA tumor tissues demonstrated that *Ihh* and *Gli2* have low expression in canine OSA tissues examined here, while there is variable and heterogeneous expression of *Ptch1* and *Gli1*.

A positive hybridization signal for canine *Smo* and *Gli2* gene expression was predominately cytoplasmic in the neoplastic cells, varying from very low to almost no signaling amongst tumor specimens. Positive hybridization signal for canine *Ihh* gene expression was predominately cytoplasmic and very heterogeneous in the neoplastic cell populations inter- and intra-tumor specimens. Many of the specimens had very low to almost no hybridization signal in most neoplastic cells; however, neoplastic cells surrounded by bone matrix were found to have a high level of positive signal. Positive hybridization signal for canine *Ptch1* and *Gli1* were predominately cytoplasmic, though some nuclear signal was noted for *Ptch1*, in the neoplastic cells. The degree of positive signal was very heterogeneous inter- and intra-tumor specimens, with regions of neoplastic cells demonstrating very high levels of positive hybridization signal to regions demonstrating very low or almost no hybridization signal. A positive hybridization signal of HH pathway components was not consistently demonstrated in the tumor microenvironment. All negative control samples lacked a positive hybridization signal. All positive control samples demonstrated high levels of, predominately cytoplasmic, signal in neoplastic and tumor microenvironment cells in canine OSA tumor tissue and normal canine brain tissue. Staining in the neoplastic cells was scored, and results were variable among samples (Table 1). However, those samples with strong expression of *Smo* mRNA also had strong mRNA expression of the other HH pathway components evaluated, whereas those with low *Smo* expression tended to have lower expression of the other pathway components. Representative examples of no staining, moderate staining, and strong staining tissues are shown in Fig 1.

**Table 1:** *In Situ* hybridization scoring of osteosarcoma samples.

Tumor Sample	Smo	Ptch1	Gli1	Gli2	Ihh
1	+	++	++	+	-
2	+++	-	+	-	+
3	-	-	-	+	-
4	+	+	-	+	-
5	+++	+++	+++	++	+
6	+++	+	++	++	-
7	+	++	+	+	+
8	++	+	-	-	-
9	++++	+++	++	++	++
10	++++	+++	-	-	-
11	++++	+++	+++	+++	+++

No staining; + <25% cells staining positive; ++ 25–50% staining; +++ 50–75% staining; ++++ >75% staining.

### Vismodegib decreases canine OSA cell proliferation *in vitro*

**Fig 2:**
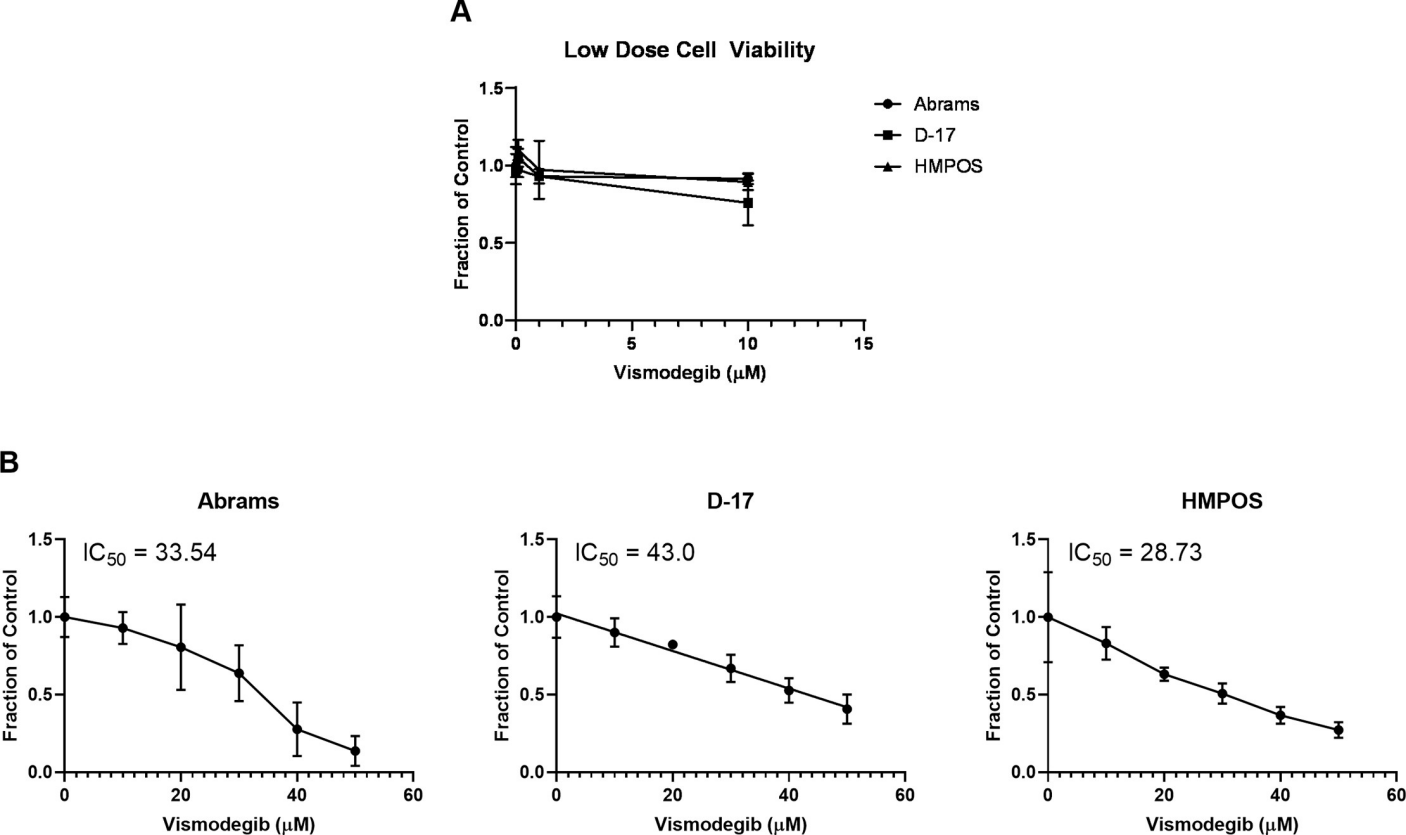
MTS cell proliferation assay. (A) Cell lines were treated with a lower and narrow dose range of vismodegib with minimal effect. (B) Abrams, D-17, and HMPOS canine OSA cell lines showed a decrease in cell proliferation with increasing concentrations of vismodegib after 72 hours of treatment. The IC_50_ at 72 hours of treatment was approximately 30 μM for Abrams and HMPOS, and 45 μM for D-17.

The next objective was to evaluate how pharmacologic inhibition of the Smo receptor affects canine osteosarcoma cells. To determine the effect of the targeted Smo inhibitor, vismodegib, on canine OSA cell viability and proliferation in relation to concentration dependent effects, we performed MTS cell proliferation assays. Abrams, D-17, and HMPOS canine OSA cell lines were treated with vehicle control (DMSO) or increasing concentrations of vismodegib and assayed at 72 hours post-treatment (Fig 2). Treatments at a lower concentration range (0.1–1.0 μM) failed to reach a 50% inhibitory effect (Fig 2A). An increased and wider dose range was used to evaluate any further effect (Fig 2B). Cell metabolic activity data was fitted to a non-linear regression model to determine the respective IC_50_ value for each cell line following 72-hours of treatment. Treatment with vismodegib resulted in a dose-dependent decrease in metabolic activity in all three canine OSA cell lines at the higher concentration range. The IC_50_ values calculated after 72 hours of treatment were approximately 30 μM of vismodegib for Abrams and HPMOS cell lines and 45 μM of vismodegib for the D-17 cell line. These concentrations were used for the remainder of the experiments.

### Vismodegib reduces canine OSA cell colony formation

**Fig 3:**
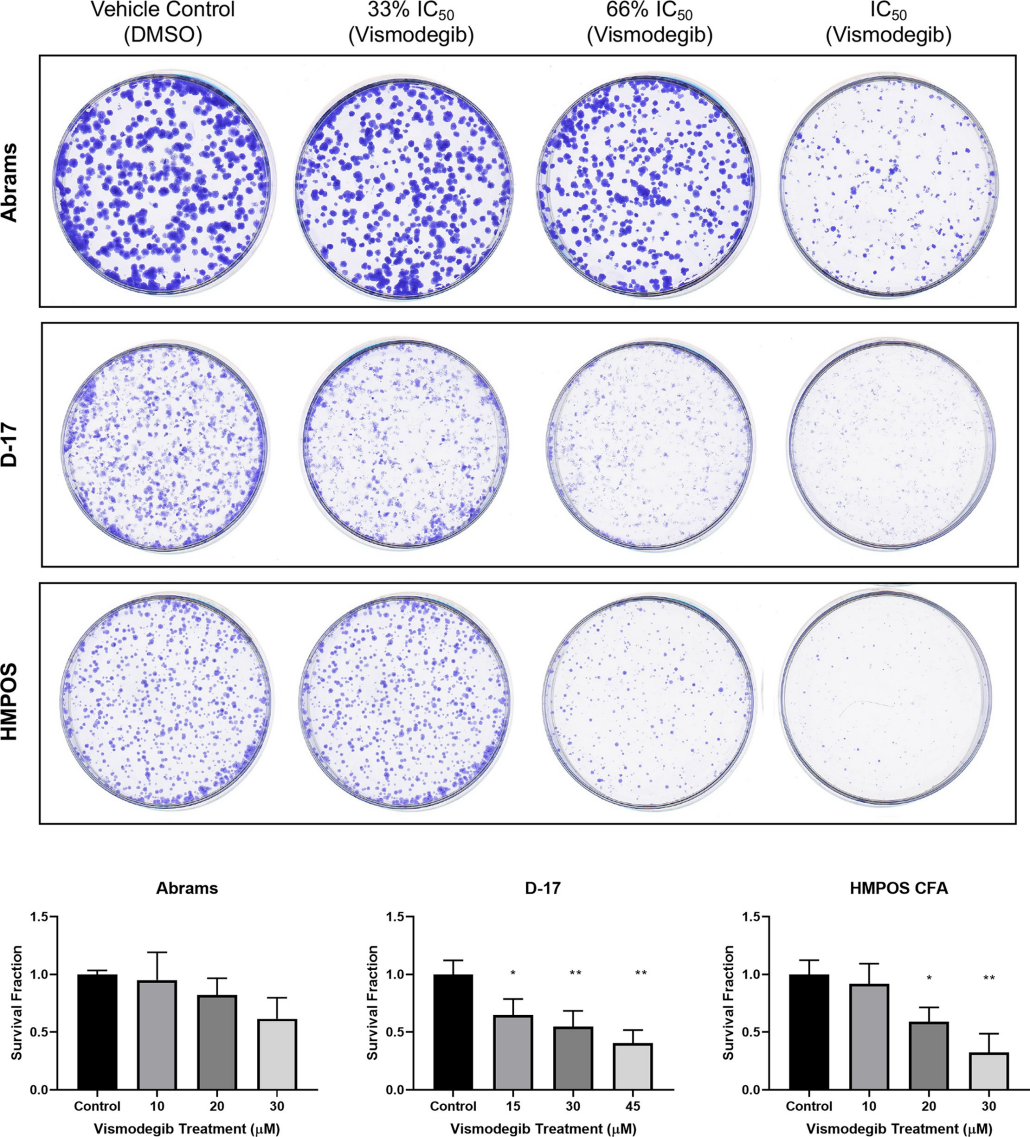
Colony formation assay. (A) Abrams, D-17, and HMPOS canine OSA cell lines treated with vehicle control (DMSO), 33% of the respective determined IC_50_ dose of vismodegib (10 μM for Abrams and HMPOS; 15 μM for D-17), 66% of the respective determined IC_50_ dose of vismodegib (20 μM for Abrams and HMPOS; 30 μM for D-17), and the full determined IC_50_ dose of vismodegib (30 μM for Abrams and HMPOS; 45 μM for D-17). Abrams, D-17, and HMPOS cell lines demonstrated decreased colony formation following treatment with vismodegib at the respectively determined IC_50_ value compared to vehicle control treatment. (B) Quantitation of colony formation assays reflected by survival fraction. * p < 0.05; **p < 0.01.

To further evaluate the effect of vismodegib on OSA cell growth and proliferation, we performed colony formation assays. Abrams, D-17, and HMPOS canine OSA cell lines were treated with vehicle control (DMSO), the determined respective IC_50_ value of vismodegib, and 33% or 67% of the respectively determined IC_50_ concentrations, for 7 days (0, 10, 20, and 30 μM for Abrams and HMPOS; 0, 15, 30, and 45 μM for D-17). Cells were fixed and stained (Fig 3A) and colonies were counted (Fig 3B) for comparison and statistical analysis. All three canine OSA cell lines were found to have reduced colony formation with vismodegib compared to vehicle control treatment, although only D-17 and HMPOS were statistically different. Agraded decrease in colony number and size with increasing vismodegib concentration (Fig 3A).

### Vismodegib does not consistently alter HH target gene expression in canine OSA cell lines

**Fig 4:**
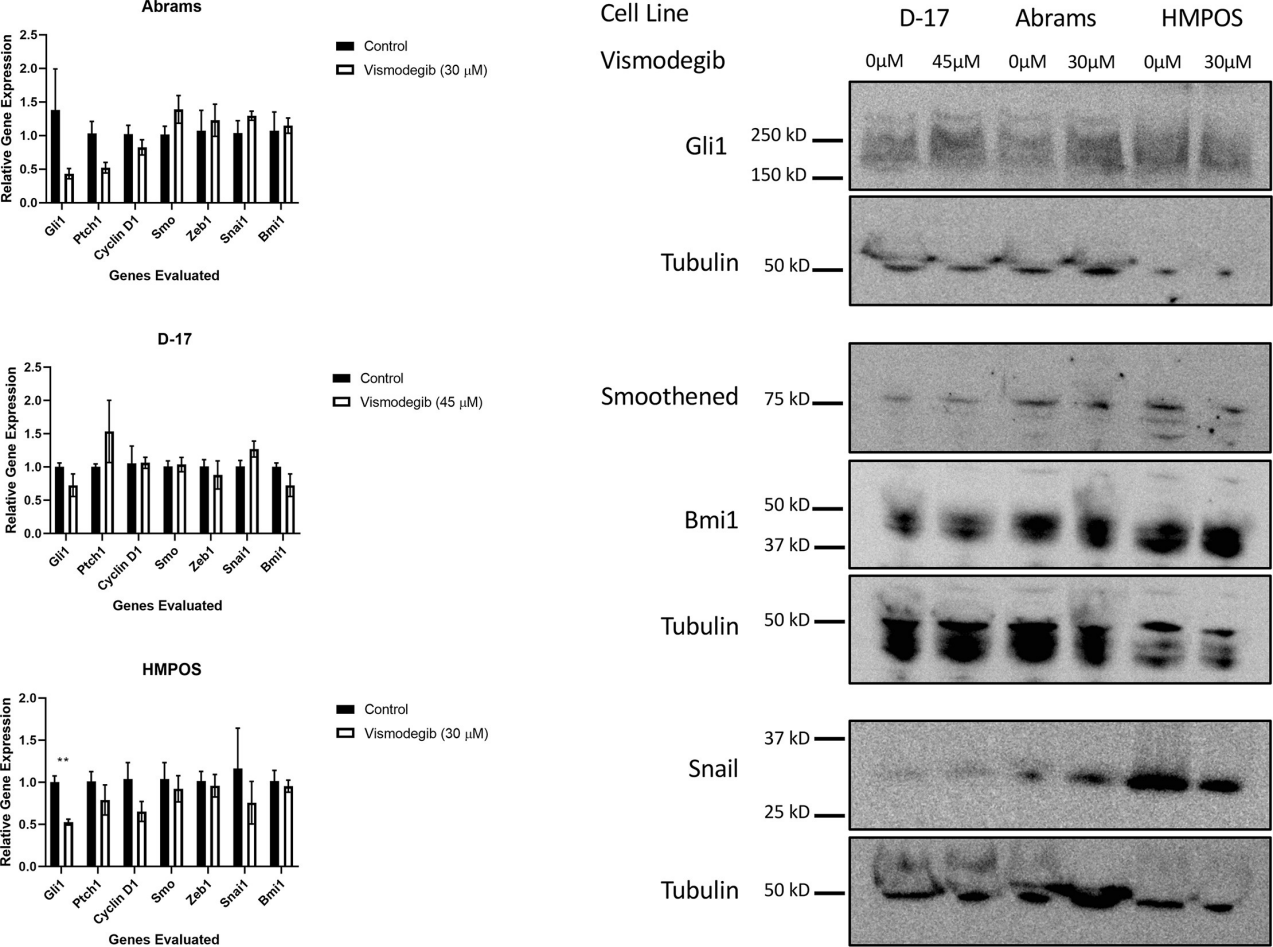
Evaluation of HH target gene mRNA and protein expression following vismodegib treatment of canine OSA cell lines. (A) The cell lines demonstrated variable decreases in some target genes at the transcript level with treatment at the determined IC_50_ value when compared to vehicle control (DMSO) treatment. HMPOS demonstrated the greatest decrease in Gli1 compared to control treated. * p <0.05. (B) Cell lines had variable expression of HH target genes when compared to each other. Treatment of Abrams, D-17, and HMPOS cells with vismodegib saw little to no change in protein expression following treatment with vismodegib. HMPOS cell appeared to demonstrate a slight decrease in Snai1 expression.

Having demonstrated that vismodegib negatively impacts canine OSA cell growth, proliferation, and viability, we investigated the effects of the drug on HH target gene expression in canine OSA cells. We evaluated genes that were direct targets of Smo-dependent Hedgehog signaling that have also been reported to be involved in osteosarcoma pathogenesis in human and canine literature. It would be expected that, if targeted Smo inhibition was occurring, a decrease in HH target gene expression would be seen. Abrams, D-17, and HMPOS canine OSA cell lines were treated with vehicle control (DMSO) or the determined respective IC_50_ value of vismodegib for 72 hours, after which RNA was harvested via Trizol isolation. The harvested RNA was used to perform RT-qPCR (Fig 4A). Target genes evaluated included canine *Gli1*, *Ptch1*, *Cyclin D1*, *Bmi1*, *Snai1*, and *Zeb1*. Canine *Smo* was also evaluated, although itself is not a direct target of the pathway. All these genes were expressed in the cell lines. Overall, the effects of treatment on the genes evaluated were variable. The most consistent change was a decrease in Gli1, although only a statistically significant decrease was seen in the HMPOS cell line. Western blot was also performed for some of these genes (Fig 4B). No consistent decrease in Gli1, Bmi1, nor Snai1 was seen. HMPOS cells did demonstrate a decrease in both the transcript and protein for Snai1. These inconsistent changes in gene expression suggest that HH pathway inhibition may contribute but does not rule out variability due to off-target effects.

To further evaluate the effect of vismodegib on some of these genes that are targets of HH signaling as well as relevant to the pathology of osteosarcoma, Western blot was also performed [24, 37–41]. The canine OSA cell lines were again treated with their respective concentrations of vismodegib and harvested after 72 hours of treatment and whole cell protein lysate collected. Targets evaluated included Gli1, Bmi1, and Snai1. Smo protein expression was also evaluated. The cell lines demonstrated variable expression of these proteins (Fig 4B). However, no consistent decreased expression of these was noted despite vismodegib treatment. HMPOS may have demonstrated a decrease in Snai1 protein expression compared to its control treated samples. Smo expression was also stable with treatment (Fig 4B). These findings support the RT-qPCR findings demonstrating a lack of clear change in HH target genes.

### Vismodegib induces apoptosis in some canine OSA cell lines

**Fig 5:**
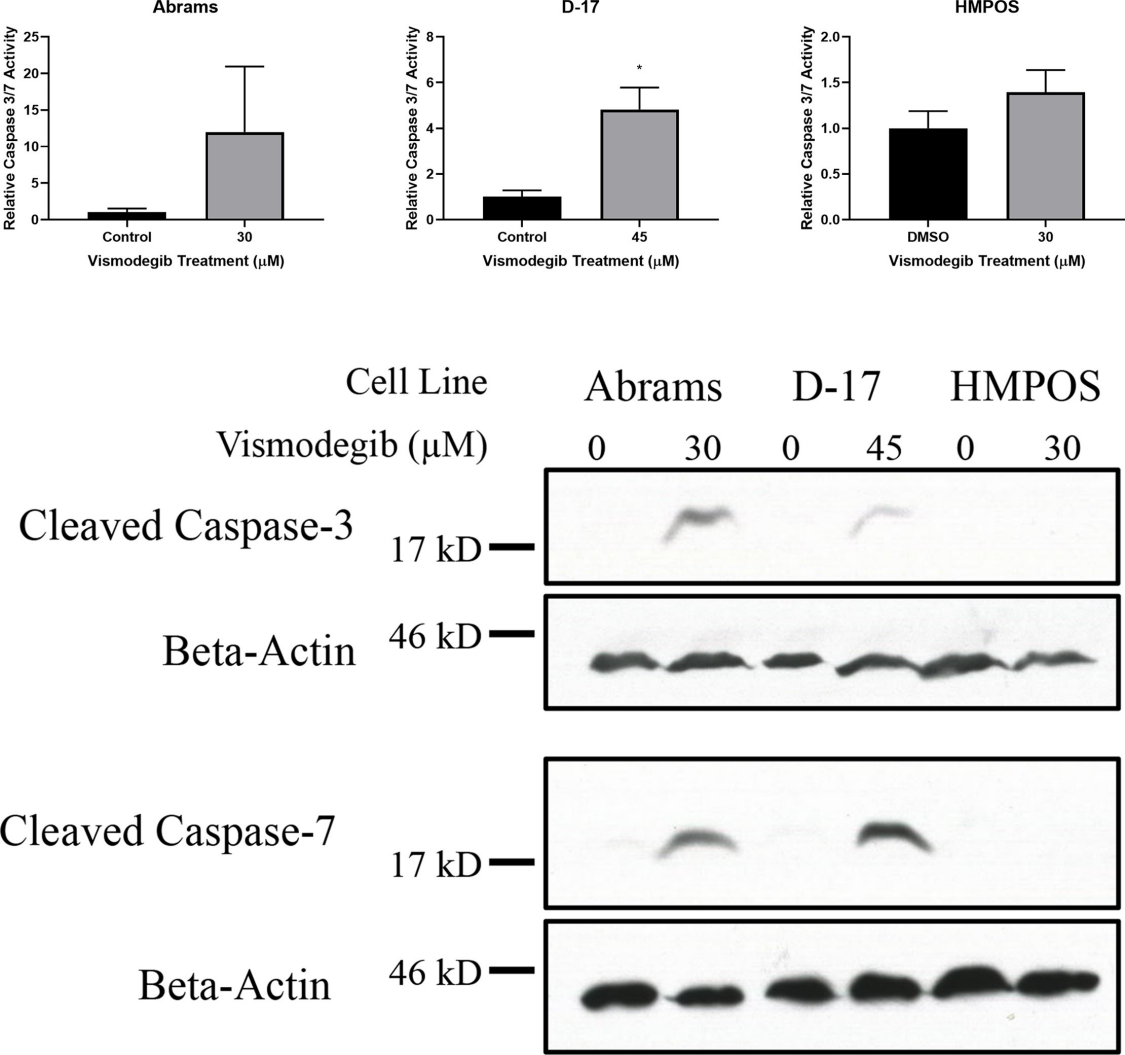
Caspase assay and caspase Western blot reveal apoptosis in canine OSA cell lines treated with vismodegib. Increased cleaved caspase 3 & 7 protein expression was detected with vismodegib treatments at the respectively determined IC_50_ value in Abrams and D-17 canine OSA cell lines when compared to vehicle control (DMSO) treatment in both (A) colorimetric caspase 3/7 assay and (B) Western blot. * p < 0.05.

We have demonstrated that vismodegib inhibits the growth, proliferation, and viability of canine OSA cell lines without consistently altering the expression of HH pathway genes or proteins. To further examine the mechanism by which vismodegib inhibits OSA cell proliferation and viability, we evaluated whether these effects were occurring due to cell death via apoptosis. Abrams, D-17, and HMPOS canine OSA cells were treated with vehicle control (DMSO) or vismodegib for 72 hours. These cells were then assessed for apoptosis using a colorimetric caspase-3/7 assay (Fig 5A). Vismodegib treatment significantly increased caspase activity in the D-17 cell line. The Abrams cell line had a substantial increase in caspase activity, but due to one replicate experiment displaying a greater increase in activity and the other two not having the same degree of increase, the total number was too low to show statistical significance because of the degree of standard deviation. There was no significant change in caspase activity in the HMPOS cell line. The results from this assay for all three cell lines were corroborated with Western blots for cleaved caspase-3 and -7.

To further assess the validity of this apoptotic finding, canine OSA cell line protein lysates described above were utilized to investigate the effect of vismodegib treatment on the expression of cleaved caspase-3 and -7 proteins via Western blot (Fig 5B). The blots revealed similar results to those obtained via caspase assay, with increased cleaved caspase-3 and -7 protein expression in Abrams and D-17 cells treated with vismodegib. No appreciable change in cleaved caspase-3 or -7 protein expression was seen in HMPOS cells between treated and control.

## Discussion

Canine osteosarcoma (OSA), the most common skeletal malignancy in dogs, is an aggressive mesenchymal malignancy of primitive bone cells with approximately 90% of patients having microscopic metastatic disease at the time of diagnosis [6, 7]. Canine OSA parallels OSA in children in numerous aspects and has been proposed as a spontaneous model for human OSA [15, 16]. As such a relevant, naturally occurring model of human cancer, comparative oncology can be used to increase our understanding of the pathways involved in canine osteosarcoma tumorigenesis and accelerate the development of additional treatment options for both species. Furthermore, because appendicular osteosarcoma is more common in dogs than in humans, research into pathogenesis and therapeutics can be conducted more rapidly and efficiently [16]. These features underscore the value of comparative oncology, particularly for osteosarcoma. The Hedgehog (HH) developmental signaling pathway is well documented to contribute to the pathogenesis of OSA in humans [22, 23]. Gene expression profiling in canine OSA identified mRNA dysregulation of *Smo*, *Ptch1*, and *Dhh* in poor responders, and a recent study found inhibition of Gli function with GANT treatment leads to decreased cell proliferation in canine OSA cell lines [15, 31]. An additional study evaluating Gli inhibition with melarsamine demonstrated similar results to the previous study evaluating GANT [42]. These studies lend support to the role of HH signaling in canine osteosarcoma. However, neither of these studies evaluated the expression patterns of HH at the cellular level in tissues nor the impact of upstream HH inhibition. The objective of our study was to evaluate the role of HH signaling inhibition through the receptor Smo, using vismodegib, an inhibitor currently available for some clinical indications in human cancer treatment.

In order to evaluate the expression of HH pathway components in canine OSA tumor tissues we performed i*n situ* hybridization for canine *Ihh*, *Smo*, *Ptch1*, *Gli1*, and *Gli2* mRNA on archived, formalin fixed, paraffin-embedded primary canine OSA samples. While our sample size was small due to the retrospective nature of evaluation, the results revealed that *Ihh* and *Gli2* mRNA have relatively low expression in canine OSA tissues examined here. We also noted that there is variable and heterogeneous expression of *Ptch1* and *Gli1* mRNA. Interestingly, *Ihh* mRNA appears to be primarily expressed in neoplastic cells producing bone matrix, with minimal to absent expression in the remaining neoplastic cells. While we are unable to further correlate these ISH findings to other clinical and pathological data due to the nature of using archival studies, these noted findings were consistent in the samples we evaluated. Additionally, the tumors that did have the highest expression of HH pathway components were also noted to be of the chondroblastic subtype of OSA. While this sample size is too small to draw conclusions regarding the implications of this finding, it is worth noting. However, the authors were unable to find published evidence that similar findings in human osteosarcoma have been made.

To the authors’ knowledge, HH pathway gene expression has not been evaluated in OSA via ISH previously. Though mRNA expression was detected, this does not necessarily correlate with the ultimate protein expression in the tissue. Further studies would be required to assess protein expression in canine OSA tumor specimens via immunohistochemistry. One current limitation of immunohistochemistry is the availability of antibodies that cross-react to the canine protein correlates in FFPE tissues. The variability of mRNA expression of HH pathway components among and between tumor specimens is suspected to correlate with the heterogeneity of tumors. The variable level of *Smo* expression likely indicates that this may not be an ideal target in the treatment of canine OSA alone, though it may play a role in combination treatments. The high levels of *Ihh* mRNA expression in neoplastic cells producing matrix is a very intriguing finding. This is consistent with data showing that IHH is expressed in osteoblast cells, playing a role in osteoblast differentiation and bone formation [21]. It may be hypothesized that inhibition of the HH signaling pathway via SMO inhibition may be a valuable component of multimodal therapy for osteoblastic OSA in dogs. Another study in human OSA demonstrated IHH expression in osteoblastic tumors via immunohistochemistry [43].

With previously published data and our ISH findings, we wanted to evaluate the effect of pharmacologic inhibition of Smo in canine osteosarcoma cell lines. With targeted inhibition of SMO, a decrease in cell viability would be expected if the HH signaling pathway were indeed active in canine OSA cells. SMO inhibition with cyclopamine, a steroidal alkaloid isolated from the *Veratrum californicum* plant, has been demonstrated in human OSA cell lines to result in a dose- and time-dependent decrease in metabolic activity [27, 29]. However, cyclopamine is a less favorable SMO inhibitor for potential *in vivo* use compared to drugs such as vismodegib due to administration challenges and toxicities that would be associated with use of cyclopamine at potentially therapeutic dosages in mice [44]. To determine the effect of the SMO inhibitor, vismodegib, on canine OSA cell viability and proliferation in relation to concentration dependent effects, we performed MTS cell proliferation assays. We demonstrated that treatment with vismodegib resulted in a dose-dependent decrease in metabolic activity in all three OSA cell lines. Investigation with lower, more pharmacologically relevant concentrations of vismodegib revealed only modest effects on cell viability. An increased and expanded dose range utilized was able to reveal a range of concentrations that resulted in an effective reduction of cell viability by at least 50%. From this, we determined D-17 cells appeared less sensitive than the Abrams or HMPOS cell lines. It is worth noting that these concentrations are indeed high, and not within a physiologically relevant range. As such, it may be that these results were due to off-target effects. To investigate this further, we performed the remainder of the experiments with the approximate IC_50_ dose calculated from this assay.

To further evaluate the effect of vismodegib on OSA cell growth and proliferation, we performed colony formation assays. We demonstrated that canine OSA cell lines D-17 and HMPOS had statistically significantly reduced colony formation with vismodegib compared to vehicle control treatment. SMO inhibition with cyclopamine has also been demonstrated in human OSA cell lines to result in a dose-dependent decrease in colony formation [29]. With targeted inhibition of SMO, these results would be expected if the HH signaling pathway were indeed active in canine OSA cells. However, similar to the MTS results, these findings may be due to off-target effects because of the dose of the inhibitor.

Having demonstrated that vismodegib inhibits canine OSA cell growth, proliferation and viability, we sought to determine whether vismodegib was acting by directly inhibiting SMO or another, off-target mechanism. It would be expected that, if targeted SMO inhibition were occurring rather than off-target effects, a decrease in HH target gene expression would be seen. We used an expanded panel of direct HH gene targets to include multiple oncogenic pathways, such as cell proliferation and metastasis. These genes included evaluation of Ptch1, Gli1, Cyclin D1, Zeb1, Snai1, and Bmi1 [37, 41]. These genes are direct targets of active HH signaling as well as have been shown to be altered in human OSA [24, 39–41]. Bmi1 is important for cell self-renewal and stem-cell like features through HH-mediated mechanisms [38, 45]. Our results, however, show that there is variable change in target gene expression. Some genes, such as Gli1, appear to decrease following vismodegib treatment in two of the three cell lines, with the HMPOS cell lines demonstrating a statistically significant decrease. However, the same thing was not found upon evaluation of protein expression with Western blot. It is important to note that the effects of HH signaling are largely through transcriptional regulation of target genes. We did not investigate other mechanisms that may affect regulation of protein expression. Hence, the greatest conclusions should be made from the RT-qPCR data assessing target gene transcription. While there are some trends suggesting an impact on HH target gene regulation, the changes following vismodegib treatment were not as robust as one might hypothesize. These results suggest that the biologic effects of vismodegib treatment may indeed be through inhibition of HH signaling, although off-target effects cannot be ruled out, particularly at the concentrations that were used in this study. Further evaluation of Smo inhibition at the genetic level, such as through knockdown experiments are needed to help characterize the dependency on Smo in canine osteosarcoma.

Having demonstrated that vismodegib inhibits canine OSA cell growth, proliferation, and viability, we evaluated if the negative impact on proliferation and viability of canine OSA cells was occurring due to cell death via apoptosis. Using a colorimetric caspase-3/7 assay, we demonstrated increased caspase activity in the D-17 cell lines with vismodegib compared to vehicle control, but not in the HMPOS cell line. The Abrams cells did demonstrate a very robust increase in caspase activity, but due to the variation in individual experiments, the statistical significance could not be demonstrated. However, all of these results and conclusions were corroborated by Western blot, which demonstrated increased cleaved caspase-3 and -7 protein expression in Abrams and D-17 cells treated at their respectively determined IC_50_ of vismodegib, while no apparent change was seen in HMPOS cells. The contrasting levels of apoptosis amongst canine OSA cell lines may represent inherent differences in the tumor from which they originated, with some differences in cells that originated from metastatic sites. There may be additional mechanisms of toxicity as well. Such findings have been demonstrated in human OSA cell lines when treated with a SMO inhibitor, with the 143B human OSA cell line demonstrating no detectable apoptosis but rather undergoing cell cycle (G_1_) arrest [27]. However, another study demonstrated significant increases in apoptosis with SMO inhibition in HOS human OSA cells [29]. Further studies would be required to assess potential differences in mechanisms or responses seen between primary and metastatic cells with SMO inhibition in canine OSA.

The findings produced here provide a platform for continued investigations regarding the role for HH signaling in canine OSA. There appears to be a dose-related toxicity in canine OSA cells. While the doses used here are higher than would be physiologically relevant, evidence still suggests a role for HH inhibition in canine osteosarcoma. As we only evaluated pharmacologic inhibition of Smo, further investigations with genetic inhibition through such experiments as Smo knockdown should be strongly considered. While homology between canine Smo and human SMO appear similar, it is possible that vismodegib interaction with canine Smo is not the same. Additionally, a combination with other therapeutics should be considered. Future directions include investigation of the combination of targeted SMO inhibition and chemotherapy, such as with carboplatin. While inhibition of canine Smo in this work did not suggest a highly sensitive target for canine OSA, previous work has showed GLI inhibition to be potentially useful [31]. Further studies investigating both canonical and non-canonical HH signaling mechanisms may clarify this discrepancy and lead to further insights to combinatorial therapies.

In conclusion, we found expression levels of Hedgehog signaling pathway component mRNA varied across primary tumor samples. Treatment of canine OSA cells with vismodegib led to a decrease in cell viability, proliferation, and survival. While some changes in downstream Hedgehog pathway target genes occurred with vismodegib treatment, this was not consistent across the cell lines. While evidence may suggest a role for Hedgehog signaling in canine osteosarcoma, further studies investigating the role for signaling through the Smoothened receptor are necessary to improve our understanding.

## Supporting information

S1 Raw images (PDF)
