# Supplementary material for: Evaluation of canonical Hedgehog signaling pathway inhibition in canine osteosarcoma
Source: PLoS One. 2020 Apr 29;15(4):e0231762. doi: 10.1371/journal.pone.0231762 (PMC7190150; doi:10.1371/journal.pone.0231762)

Figure 4B: Raw Data
- Canine OSA cell lines treated with vismodegib
- Image captured by chemiluminescence detection via digital imaging with Kodak Image Station

Raw Image for Figure 4B: Gli1 blot
Anti-Gli1 antibody
Abcam #49314
1:500 dilution, ~160 kDa

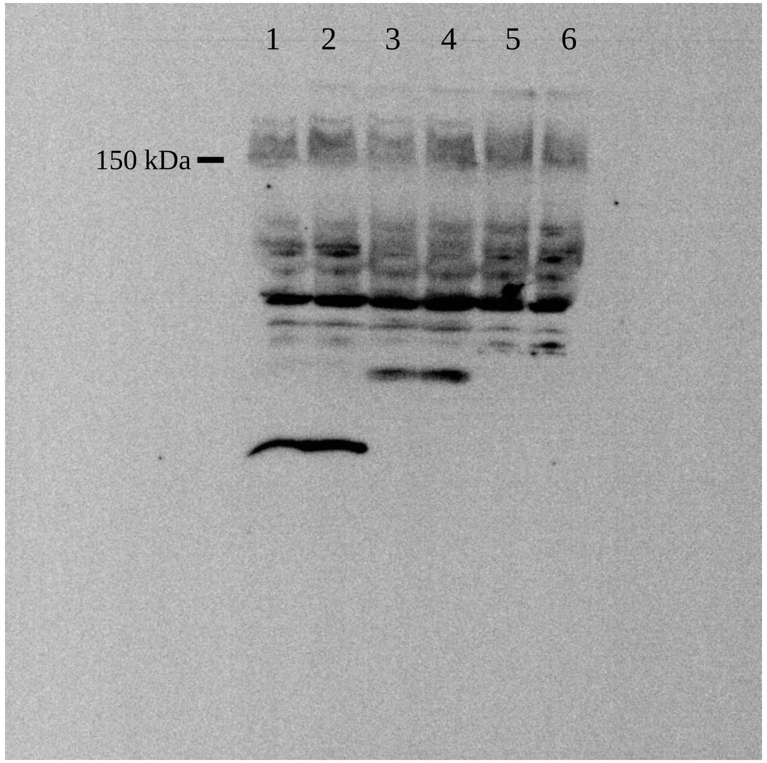

Raw Image for Figure 4B:
Tubulin Loading Control for Gli1 blot
Sigma #T9026
1:5000 dilution, ~50 kDa

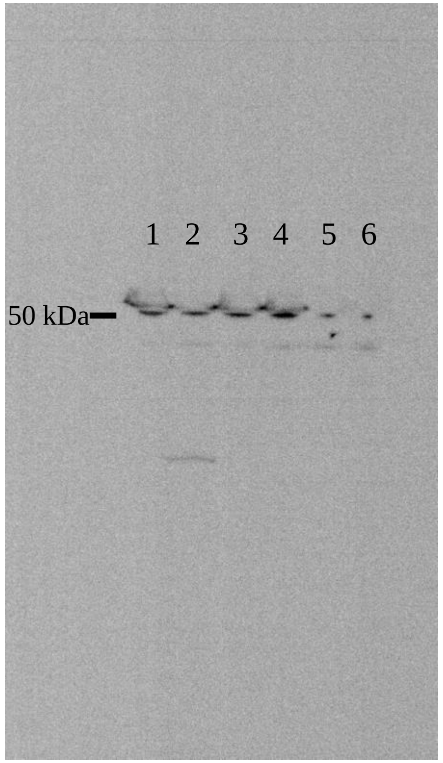

Key:
1. D-17 whole cell lysate, DMSO control treated
2. D-17 whole cell lysate, 45 µM vismodegib treated
3. Abrams whole cell lysate, DMSO control treated
4. Abrams whole cell lysate, 30 µM vismodegib treated
5. HMPOS whole cell lysate, DMSO control treated
6. HMPOS whole cell lysate, 30 µM vismodegib treated

Figure 4B: Raw Data

- Canine OSA cell lines treated with vismodegib
- Image captured by chemiluminescence

detection via digital imaging with Kodak Image Station

Raw Image for Figure 4B: Smo blot
Anti-Smo antibody
Abcam #ab236456
1:500 dilution, ~86 kDa

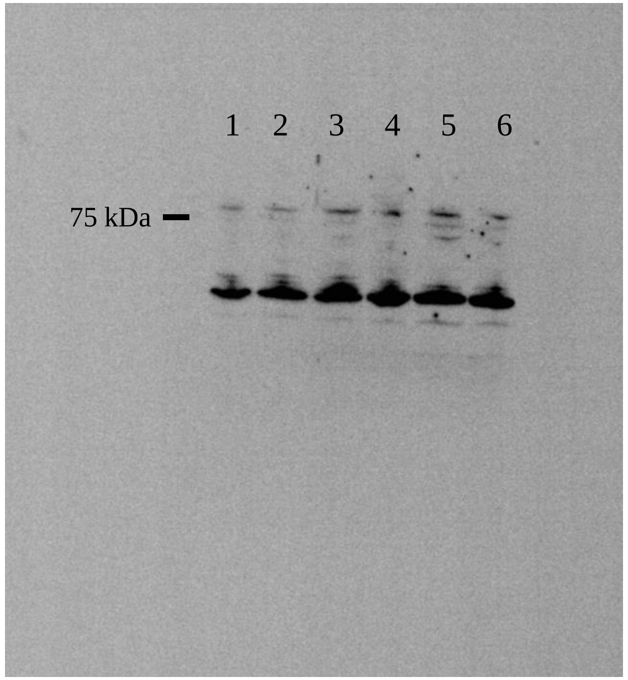

Raw Image for Figure 4B: Bmi1 blot
Anti-Bmi1 antibody
Cell Signaling #6964
1:2000 dilution, ~45 kDa

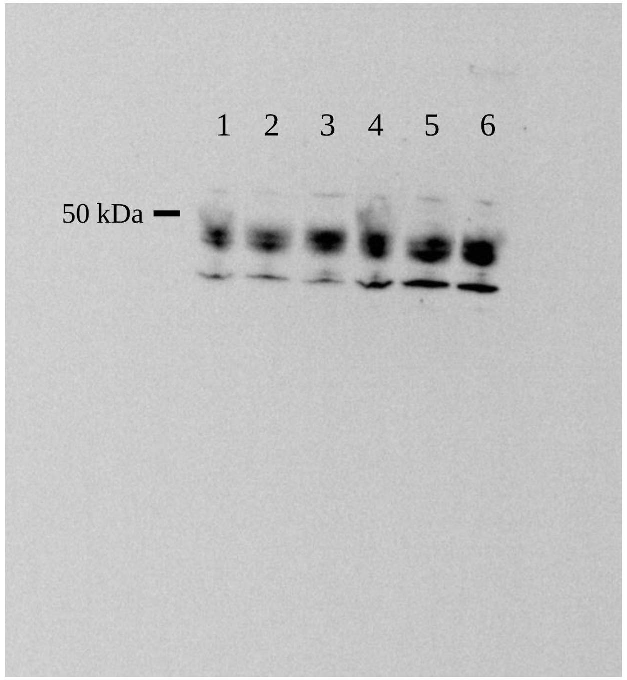

Raw Image for Figure 4B:
Tubulin Loading Control for Smo/Bmi1 blot
Sigma #T9026
1:5000 dilution, ~50 kDa

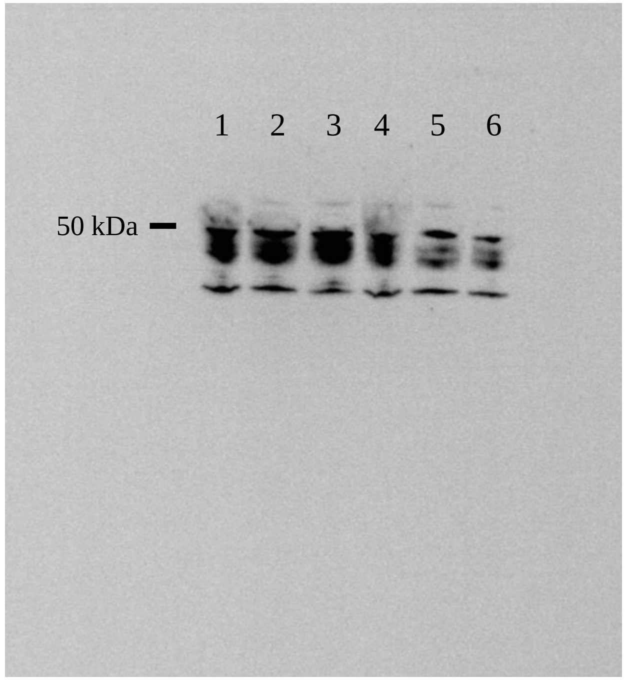

Key:

1. D-17 whole cell lysate, DMSO control treated
2. D-17 whole cell lysate, 45 µM vismodegib treated
3. Abrams whole cell lysate, DMSO control treated
4. Abrams whole cell lysate, 30 µM vismodegib treated
5. HMPOS whole cell lysate, DMSO control treated
6. HMPOS whole cell lysate, 30 µM vismodegib treated

Figure 4B: Raw Data
- Canine OSA cell lines treated with vismodegib
- Image captured by chemiluminescence detection via digital imaging with Kodak Image Station

Raw Image for Figure 4B: Snai1 blot
Anti-Snai1 antibody
Cell Signaling #3879
1:500 dilution, ~29 kDa

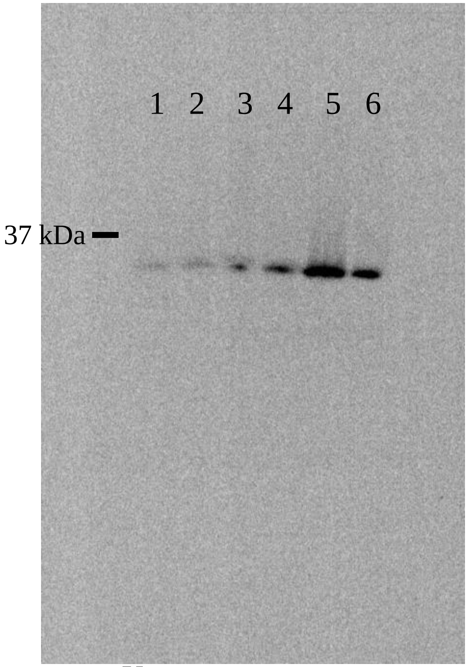

Raw Image for Figure 4B:
Tubulin Loading Control for Gli1 blot
Sigma #T9026
1:5000 dilution, ~50 kDa

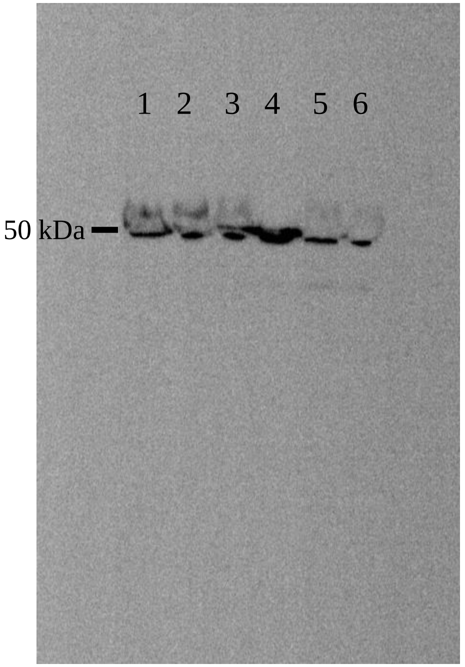

Key:
1. D-17 whole cell lysate, DMSO control treated
2. D-17 whole cell lysate, 45 µM vismodegib treated
3. Abrams whole cell lysate, DMSO control treated
4. Abrams whole cell lysate, 30 µM vismodegib treated
5. HMPOS whole cell lysate, DMSO control treated
6. HMPOS whole cell lysate, 30 µM vismodegib treated

Raw Image for Figure 4B: Cleaved Caspase-3 blot
Anti-Cleaved Caspase-3 antibody
Cell Signaling #9661
1:1000 dilution, ~19 kDa

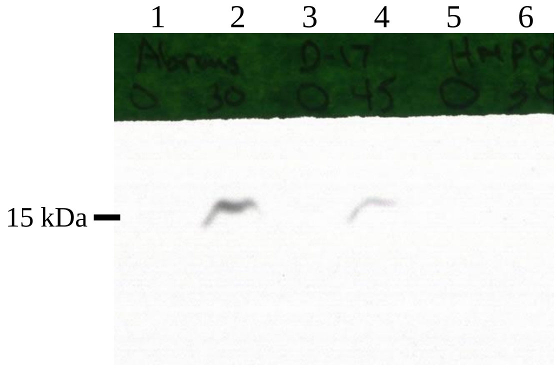

Raw Image for Figure 4B:
Tubulin Loading Control for Smo/Bmi1 blot
Sigma #T9026
1:5000 dilution, ~50 kDa

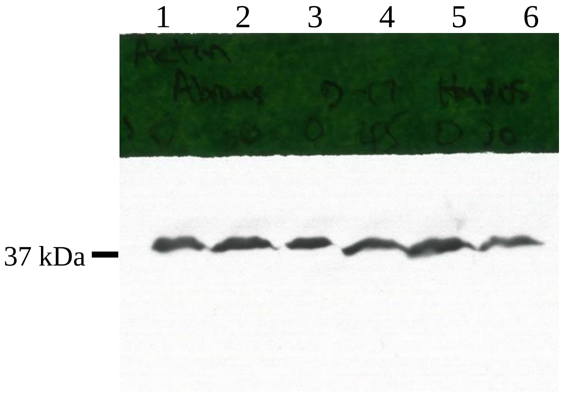

Figure 5B: Raw Data

- Canine OSA cell lines treated with vismodegib
- Image captured by chemiluminescence detection via radiographic film exposure; films scanned

Key:

1. Abrams whole cell lysate, DMSO control treated
2. Abrams whole cell lysate, 30 µM vismodegib treated
3. D-17 whole cell lysate, DMSO control treated
4. D-17 whole cell lysate, 45 µM vismodegib treated
5. HMPOS whole cell lysate, DMSO control treated
6. HMPOS whole cell lysate, 30 µM vismodegib treated

Raw Image for Figure 4B: Cleaved Caspase-7 blot
Anti-Cleaved Caspase-7 antibody
Cell Signaling #8438
1:1000 dilution, ~18 kDa

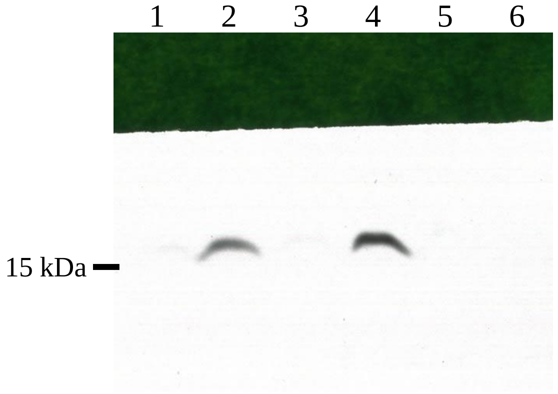

Raw Image for Figure 4B:
Tubulin Loading Control for Smo/Bmi1 blot
Sigma #T9026
1:5000 dilution, ~50 kDa

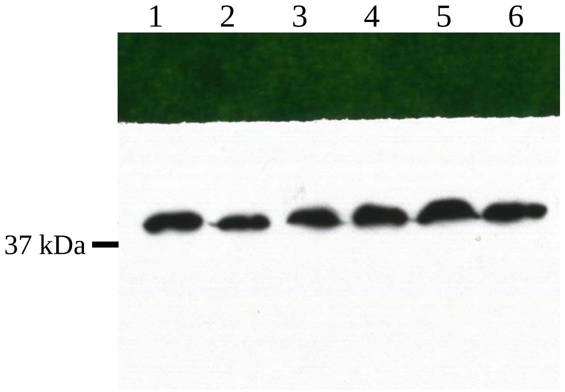

Supplement: S1 Raw images — (PDF) [file pone.0231762.s001.pdf]
